# Prescribing Patterns of High Opioid and Antibiotic Prescribers, Washington State, 2021: Do Some Prescribers Have Trouble Saying No?

**DOI:** 10.1093/ofid/ofae657

**Published:** 2024-11-05

**Authors:** David Trey Evans, Katarina Kamenar, Jessica Zering, Marisa D’Angeli, Erica J Stohs

**Affiliations:** Healthcare Associated Infections Program, Washington State Department of Health, Shoreline, Washington, USA; Healthcare Associated Infections Program, Washington State Department of Health, Shoreline, Washington, USA; Healthcare Associated Infections Program, Washington State Department of Health, Shoreline, Washington, USA; Healthcare Associated Infections Program, Washington State Department of Health, Shoreline, Washington, USA; Healthcare Associated Infections Program, Washington State Department of Health, Shoreline, Washington, USA; Division of Infectious Diseases, Department of Medicine, University of Nebraska Medical Center, Omaha, Nebraska, USA

**Keywords:** antimicrobial stewardship, opioids, physician prescribing pattern

## Abstract

Among Washington State emergency and family medicine physicians, high prescribers of opioids were 2.9 times more likely to be high prescribers of antibiotics in the Medicare Part D population. The inverse relationship showed the same association. Antimicrobial and opioid stewards should collaborate on shared goals to implement effective interventions.

Antibiotic resistance and the opioid epidemic are critical public health threats in the United States needing comprehensive intervention strategies [1, 2]. The Centers for Disease Control and Prevention attribute approximately 36 000 deaths per year to antibiotic resistance [3] and more than 51 000 deaths per year to opioid overdose [4]. Heightened focus on prescribing in their respective fields led to overlapping quality and safety initiatives nationally. Opioid and antimicrobial stewardship share many of the same tenants: leadership commitment; drug expertise; tracking, monitoring and reporting; accountability; and education of clinicians, patients and families [5, 6].

Previous analyses from the Department of Veteran Affairs (VA) system and the National Health Service in the United Kingdom found notable associations between rates of opioid and antibiotic prescribing among dental and medical providers [7, 8]. We explored this relationship in a broader primary care context in Washington State (WA) using Centers for Medicare & Medicaid Services Medicare Part D (MPD) data. In this study, we sought to determine if an association between high antibiotic and opioid prescribing existed among emergency department and family medicine physicians. Secondarily, we sought to measure how this association was influenced by provider characteristics.

## METHODS

MPD insurance covers approximately 70% of the United States population aged ≥65 years and persons with disabilities or end-stage renal disease [9]. This analysis used the publicly available 2021 Centers for Medicare & Medicaid Services MPD Prescribers by Provider file released in June 2023 to assess providers’ antibiotic and opioid prescribing practices. This dataset quantifies outpatient prescriptions at the prescriber level for 3 categories of drugs (antibiotics, antipsychotics, and opioids), alongside provider details such as provider type, sex, and practice address [9]. Family medicine and emergency medicine physicians were included if the following criteria were met: a practice address in WA, licensed in WA, and record of prescribing at least 1 antibiotic and 1 opioid during the study period. Nurse practitioners, physician assistants, and internal medicine physicians were excluded from this analysis because there was no available data defining their practice area (eg, emergency medicine, primary care).

A previous analysis of MPD data defined a high antibiotic prescriber as a provider prescribing in the top 10th percentile based on their volume of antibiotics prescribed [9]. In this study, high prescribing behavior was defined as prescribing at a rate, drug claims per 1000 beneficiaries, ≥90th percentile of their specialty (*Z*-score ≥ 1.282). Individual physician prescribing rates for antibiotics and short-acting opioids were calculated. Physicians were stratified by specialty—family medicine and emergency medicine. Prescribing rates for both opioid and antibiotics were converted to *Z*-scores, which allowed us to combine the observations from both groups in the model while accounting for a physician's specialty. Multivariate logistic regression was used to model the odds of being a high prescriber of antibiotics as a function of providers’ opioid prescribing behavior and vice versa. Models were adjusted for physician age and sex. Physician age was obtained from the WA Department of Health's credential database. Analyses were conducted in R software (version 4.3.1; R Foundation).

## RESULTS

### Physician Characteristics

In 2021, a total of 2766 family medicine and emergency medicine physicians prescribed both opioids and antibiotics to MPD beneficiaries (see Table 1). Median age of the physicians was 48 years (interquartile range [IQR]: 39–56) and 57% were male. The median prescribing rate for antibiotics was 212 (IQR: 134–397) antibiotic claims per 1000 beneficiaries and the median prescribing rate for opioids was 328 (IQR: 204–583) opioid claims per 1000 beneficiaries. Three hundred and eight physicians (11%) were identified as high prescribers of antibiotics and 244 physicians (9%) were identified as high prescribers of opioids.

**Table 1.** Characteristics of Emergency Medicine and Family Practice Physicians (n = 2766)

Characteristics	Level	Emergency Medicine	Family Practice
N	…	603	2163
Sex (%)	Female	138 (22.9)	1044 (48.3)
	Male	465 (77.1)	1119 (51.7)
High antibiotic prescriber (%)	…	65 (10.8)	243 (11.2)
High opioid prescriber (%)	…	40 (6.6)	204 (9.4)
Age (median [interquartile range])	…	47.0 [40.0–54.0]	48.0 [38.0–57.0]

Medicare Part D, Washington State, 2021.

### Multivariate Analysis

Table 2 shows the likelihood of being a high antibiotic prescriber as a function of age, sex, and high opioid prescribing behavior. The unadjusted odds of being a high antibiotic prescriber were 3.58 times (95% confidence interval [CI], 2.61–4.87, *P* < .001) greater among physicians who were high prescribers of opioids. After adjustment, the overall odds of being a high antibiotic prescriber was 2.91 times (2.10–4.00; *P* < .001) greater among physicians who were high prescribers of opioids, 1.51 times (95% CI, 1.16–1.97) greater among male compared to female physicians, and 1.27 times (95% CI, 1.14–1.42; *P* < .001) greater for each 10-year increase in provider age.

**Table 2.** Odds of Being a High Prescriber by Prescriber Characteristics: Family Practice and Emergency Medicine Physicians (n = 2766), Medicare Part D, Washington State, 2021

Characteristic	Unadjusted			Adjusted		
	OR	95% CI	*P*	OR	95% CI	*P*
2A. Odds of Being a High Prescriber of Antibiotics						
Age (10-year increments)	1.42	1.27–1.57	<.001	1.27	1.14–1.42	<.001
Sex	…	…	…	…	…	…
Female	…	…	…	…	…	…
Male	1.84	1.43–2.38	<.001	1.51	1.16–1.97	.003
High opioid prescriber	3.58	2.61–4.87	<.001	2.91	2.10–4.00	<.001
2B. Odds of Being a High Prescriber of Opioids						
Age (10-year increments)	1.76	1.57–1.99	<.001	1.63	1.44–1.85	<.001
Sex						
Female	…	…	…	…	…	…
Male	1.88	1.42–2.51	<.001	1.31	.97–1.77	.083
High antibiotic prescriber	3.58	2.61–4.87	<.001	2.9	2.09–3.99	<.001

Table 2A: Null deviance = 1933; Null df = 2765; Log-likelihood = −945, AIC = 1894; BIC = 1577; Deviance = 1,890, Residual df = 2764. Table 2B: Null deviance = 1651; Null df = 2765; Log-likelihood = −780, AIC = 1565; BIC = 1577; Deviance = 1,561, Residual df = 2764.

Abbreviations: CI, confidence interval; OR. odds ratio.

The likelihood of being a high opioid prescriber as a function of age, sex, and high antibiotic prescriber behavior is shown in Table 2. The unadjusted odds of being a high opioid prescriber were 3.58 times (95% CI, 2.61–4.87; *P* < .001) greater if the provider was also a high antibiotic prescriber. After adjusting for age and sex, the odds of being a high opioid prescriber were 2.90 times (2.09–3.99, *P* < .001) greater among physicians who were high antibiotic prescribers. Similar to the first model, the adjusted odds of being a high opioid prescriber increased as age of the provider increased; 1.63 times (95% CI, 1.44–1.85; *P* < .001) greater odds of being a high prescriber for each 10-year increase. After adjusting for age and being a high prescriber of antibiotics, there was no significant association between the sex of the provider and being a high prescriber of opioids (odds ratio = 1.31; 95% CI, .97–1.77; *P* > .05).

## DISCUSSION

A statistically significant association was observed between high antibiotic and high opioid prescribing rates among emergency and family medicine physicians. These results suggest that “high” prescribing of medications is a provider behavior, consistent with findings from studies conducted in different practice contexts [7, 8, 10, 11]. This analysis was a novel approach using MPD data at the state level to investigate provider prescribing patterns.

Previous analyses examining potential association between high opioid and antibiotic prescribing rates in primary care settings were set within the VA, the United Kingdom's National Health Service, and in Ontario, Canada, but to date no analysis has examined primary care antibiotic and opioid prescribing in the broader US healthcare system [7, 8, 10]. A national cross-sectional study of the VA primary care population found a similar adjusted odds ratio (2.87) of medical providers being high prescribers of antibiotics and opioids [7]. Although previous analyses focused on additional factors such as practice factors and geographical location [7, 10, 12], ours focused only on prescriber characteristics to better understand provider-specific behaviors. A strength of this analysis is its focus on family and emergency medicine physicians who serve a diverse patient population and cover multiple health systems.

Increased provider age was associated with higher rates of opioid and antibiotic prescribing. Findings from previous studies among providers serving the Veterans’ Health administration that included age of the provider aligned with these results [7, 12]. A systematic review previously demonstrated that physician performance declines over time, as they become less receptive to new standards of care [13]. This association underscores the necessity for targeted outreach and ongoing education to align clinical practices with updated prescribing guidelines. In contrast to previous studies [7, 12], we did not find a consistent difference in high prescribing between males and female physicians. This inconsistency could be due to differences in how “high prescribing” was defined between studies or underlying differences in the provider population.

Traditionally, opioid and antimicrobial stewardship have been treated as separate domains despite the shared goal of enhancing patient safety and public health. Opportunities exist for stewards in both disciplines to learn from each other such as uptake of clinical decision support tools to optimize prescribing [14, 15], shorter drug durations for acute conditions [16, 17, 18], optimizing discharge prescribing [19, 20], and communication strategies regarding benefits and risks of drugs and contingency planning [17, 21, 22]. Additionally, global factors such as physician burnout and the relationship between antibiotic and opioid prescribing with patient satisfaction are ongoing challenges worth addressing [23, 24, 25, 26].

The findings of this report are subject to limitations. First, the data were limited to WA physicians practicing in family and emergency medicine and thus may not be applicable to other disciplines and provider populations. This analysis could be replicated in other states or at the national level to determine potential regional differences in prescribing. Second, we were unable to account for potential differences in prescribing in rural versus urban areas because MPD datasets attribute a provider's entire prescribing to a single address, and we were unable to ascertain if the providers were practicing at multiple locations or via telemedicine. Third, internal medicine physicians and advanced practice practitioners were unable to be included because of the inability to associate these providers with a particular primary care setting. Fourth, these results may not be generalizable to patients of all ages because MPD data only covers patients older than age 65 years and those with certain health conditions. Fifth, we were unable to account for patient characteristics’ influence prescribing rates because MPD contains limited patient population information. Finally, we could not assess the appropriateness of prescribing in our analysis.

In summary, these results suggest that “high” prescribing of medications is a provider behavior, and there are opportunities to improve judicious use of opioids and antibiotics. Next steps for this work might include partnerships or workgroups to explore synergistic structural and procedural interventions to optimize medication prescribing and reduce patient harm.

## Notes

***Author Contributions.*** D.T.E.: Methodology, formal analysis, writing—original draft, visualization. K.K.: methodology, formal analysis, writing—review & editing, visualization. J.Z.: writing—review & editing. M.D.: conceptualization, writing—review & editing. E.J.S.: writing—review & editing, supervision.

***Acknowledgments***. None

***Financial support***. None.

***Patient consent.*** No patient information was included in the data analyzed. Datasets used in the analysis are public use files compiled by Centers of Medicare and Medicaid Services. The design of the work conforms to standards currently applied by the Washington State Department of Health, Division of Communicable Disease Epidemiology in the United States.
